# Supplementary material for: Alumina Ceramic Exacerbates the Inflammatory Disease by Activation of Macrophages and T Cells
Source: Int J Mol Sci. 2020 Sep 26;21(19):7114. doi: 10.3390/ijms21197114 (PMC7583733; doi:10.3390/ijms21197114)
Supplement: Supplementary file 1 [file ijms-21-07114-s001.pdf]

International Journal of
Molecular Sciences

MDPI

Supporting information for

Alumina Ceramic Exacerbates the Inflammatory Disease by Activation of Macrophages and T cells

Seong-Min Lim[1,2,3], Juyoung Hwang[1,2,3], Hae-Bin Park[1,2,3], Chan Ho Park[4,*] and Jun-O Jin[1,2,3*]

1 Shanghai Public Health Clinical Center & Institutes of Biomedical Sciences, Shanghai Medical College, Fudan University, Shanghai 201508, China
2 Department of Medical Biotechnology, Yeungnam University, Gyeongsan 38541, South Korea
3 Research Institute of Cell Culture, Yeungnam University, Gyeongsan 38541, South Korea
4 Department of Orthopedic Surgery, Yeungnam University Medical Center, 170, Hyeonchung-ro, Nam-gu, Daegu 42415, South Korea.
* Correspondence: Chan Ho Park, orthoparkch@yu.ac.kr; Tel.:+82-053-620-3643 and Jun-O Jin, jinjo@yu.ac.kr; Tel:+82-053-810-3033

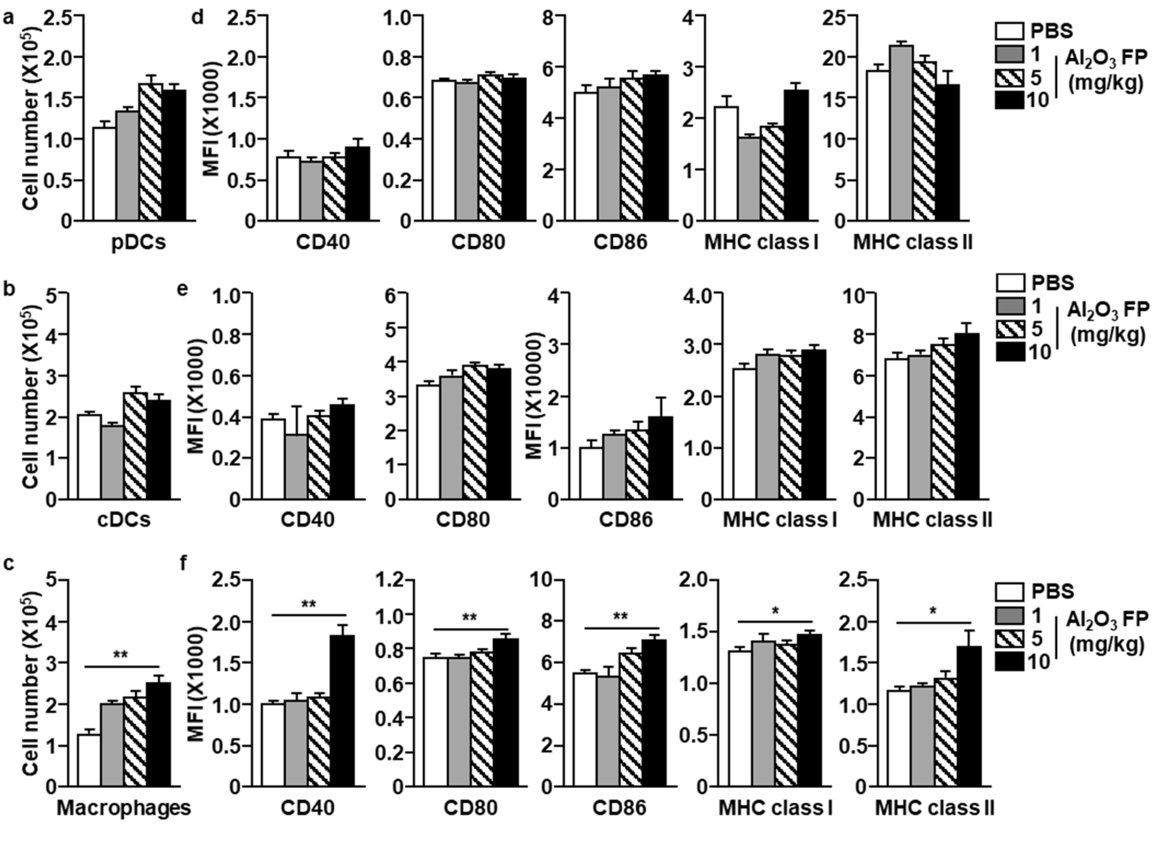

Figure S1. Dose dependent effect of Al_2O_3 FPs in the activation of innate immune cells. C57BL/6 mice were *i.p.* injected with 1, 5 and 10 mg/kg of Al_2O_3 FPs. Six hours after injection of Al_2O_3 FPs, spleen was harvested and analyzed co-stimulators and MHC class I and II expression. The absolute numbers of (a) pDCs, (b) cDCs and (c) macrophages in the spleen. The expression levels of co-stimulator and MHC class I and II were shown in (d) pDCs, (e) cDCs and (f) macrophages.

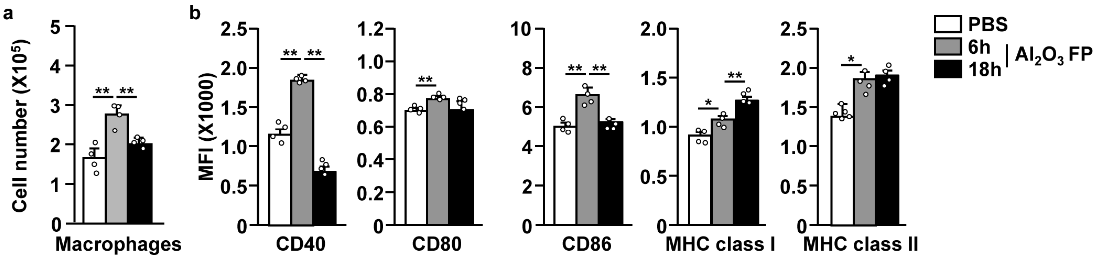

Figure S2. Time-dependent effect of Al_2O_3 FPs in macrophage activation. C57BL/6 mice were *i.p.* injected with 10 mg/kg of Al_2O_3 FPs. Six and eighteen hours after injection, the macrophage activation was analyzed. (a) The absolute number of macrophages analyzed in the spleen. (b) The expression levels of co-stimulator and MHC class I and II were shown in macrophages. The data are averages of four independent samples (2 mice for two experiments, two-way ANOVA, mean ± SEM).

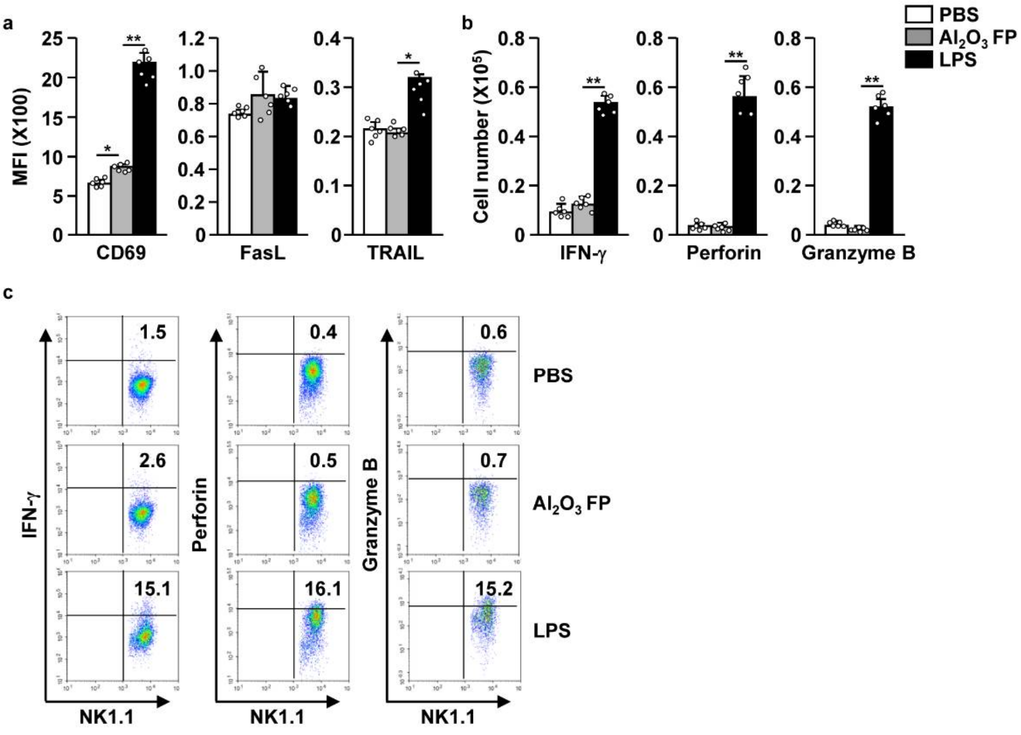

Figure S3. Al_2O_3 FPs did not induce the activation of NK cells in the mice. C57BL/6 mice were *i.p.* injected with 10 mg/kg of Al_2O_3 FPs for 6 h. The NK cells were defined as $CD3^-NK1.1^+$ cells in live leukocytes as shown in figure 2b. (a) The expression levels of CD69, Fas ligand (FasL), and TNF-related apoptosis-inducing ligand (TRAIL) are shown in NK cells as mean fluorescence intensity (MFI). (2 mice pre 3 experiments, n = 6). (b) The number of IFN-γ-, Perforin-, and Granzyme B-producing NK cells. (c) The percentages of IFN-γ-, Perforin-, and Granzyme B-producing NK cells (2 mice per 3 experiments, n = 6).

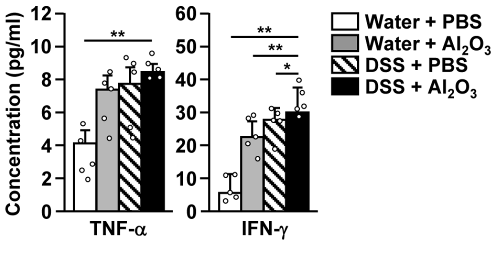

Figure S4. The serum concentrations of TNF-α and IFN-γ. The mice were treated as indicated in figure 5. The serum concentrations of TNF-α and IFN-γ were measured in triplicates by ELISA.
